# Sickle-Cell Disease Healthcare Cost in Africa: Experience of the Congo

**DOI:** 10.1155/2016/2046535

**Published:** 2016-02-02

**Authors:** L. O. Ngolet, M. Moyen Engoba, Innocent Kocko, Alexis Elira Dokekias, Jean-Vivien Mombouli, Georges Marius Moyen

**Affiliations:** ^1^Clinical Hematology Unit, Brazzaville Teaching Hospital, Auxence Ickonga Avenue, P.O. Box 32, Brazzaville, Congo; ^2^Pediatric Intensive Care Unit, Brazzaville Teaching Hospital, Brazzaville, Congo; ^3^National Laboratory of Public Health, Brazzaville Teaching Hospital, Brazzaville, Congo

## Abstract

*Background*. Lack of medical coverage in Africa leads to inappropriate care that has an impact on the mortality rate. In this study, we aimed to evaluate the cost of severe acute sickle-cell related complications in Brazzaville.* Methods*. A retrospective study was conducted in 2014 in the Paediatric Intensive Care Unit. It concerned 94 homozygote sickle-cell children that developed severe acute sickle-cell disease related complications (average age 69 months). For each patient, we calculated the cost of care complication.* Results*. The household income was estimated as low (<XAF 90,000/<USD 158.40) in 27.7%. The overall median cost for hospitalization for sickle-cell related acute complications was XAF 65,460/USD 115.21. Costs were fluctuating depending on the generating factors of the severe acute complications (*p* = 0.041). They were higher in case of complications generated by bacterial infections (ranging from XAF 66,765/USD 117.50 to XAF 135,271.50/USD 238.07) and lower in case of complications associated with malaria (ranging from XAF 28,305/49.82 to XAF 64,891.63/USD 114.21). The mortality rate was 17% and was associated with the cost of the case management (*p* = 0.006).* Conclusion*. The case management cost of severe acute complications of sickle-cell disease in children is high in Congo.

## 1. Introduction

Sickle-cell disease is the most frequent haemoglobin disorder in the world, mostly in sub-Saharan Africa where 75% of the 300, 000 babies are born each year with haemoglobin disorders live [1, 2]. Homozygous form of SCD is associated with very high child mortality, but reliable data are lacking. WHO estimates that 70% of deaths are avoidable by putting in place “preventive” measures [3]. These measures encompass early diagnosis, information, education, and prophylaxis of infections [4–6]. In sub-Saharan Africa, medical coverage program like Medicaid that supports patients in the expenses generated by medical management of their disease does not exist or is very insufficient. In Congo where 1% of the population is affected by the homozygous form of the disease, such program does not exist [7]. The nonaccessibility to care and medicines is linked in 54.61% to financial challenges [8]. This inaccessibility is higher at the tertiary level of medical facilities such as the teaching hospital where the costs of care are far higher. The consequence of this is the arrival of patients at an advanced stage of the disease. Overall, in Africa and in Congo, contrary to the Western countries, morbidity and mortality are associated with the costs of sickle-cell disease (SCD) care. Although hospitalization appears to drive the majority of SCD treatment cost [9–11], only one study published in Africa has examined the hospitalization cost of SCD [12]. That small study was conducted on adult in the Hematology Unit in Congo in 2012. None studies report the cost of care for SCD children inpatient hospitalization. The purpose of this study is to report the cost of severe acute complications of SCD related care for pediatric inpatients hospitalization in the Emergency Department of the Brazzaville Teaching Hospital in Congo.

## 2. Material and Methods

Brazzaville Teaching Hospital has a total of 4 pediatric departments: Intensive Care, Neonatology, Infant Care, and Toddler/Teenager care where a total of 5,657 children were admitted during the period of our study. This study was conducted from January 1 to December 31, 2014, in the Pediatric Intensive Care Unit (PICU) of Brazzaville Teaching Hospital. PICU manages all medical emergencies that involve immediate vital prognostic of children aged 1 month to 16 years old.

### 2.1. Data Collection and Assessment Procedures

We enrolled in the study all pediatric patients that were developing severe acute SCD related complications. SCD children with no severe SCD related complications were not included in the study. We defined as severe acute complications all life threatening sickle-cell disease related acute complications. These are

1. acute exacerbation of anemia due to acute splenic sequestration or acute hyperhemolysis;
2. major acute pain syndromes;
3. acute chest syndrome;
4. stroke;
5. mixed severe acute crisis that combines acute exacerbation of anemia and major pain syndromes;
6. severe infection: in the context of functional asplenia, a temperature over 38, 5°C with or without a source is an emergency and considered as severe infection.

*Generated Factors*. Generated factors are factors that trigger acute exacerbation anemia, pain syndromes, acute chest syndrome, stroke, and mixed crisis. These factors are infectious or physical such as environmental heat, stress, wearing heat retaining clothing, and dehydration.

Thus, 94 sickle-cell disease children presenting one severe acute complication have been included in the study.

### 2.2. Cost Assessment of the Case Management of Severe Acute Complications

Costs generated correspond to the fees encountered for the diagnosis and treatment of acute complications and generating factors. The global cost was calculated by adding costs of medicines, hospitalization and diagnosis tests.

Hospitalization fees depend on the length of admission of the patient. Patients are charged XAF 25,000/USD 42.64 for the total of 5 days (first five days at the hospital) and then XAF 5,000/USD 11.72 for each day from the 6th day of hospitalization. Costs of medicines and diagnostic tests have been calculated from the fares displayed by the teaching hospital. The consumables, dressing, syringes, and infusion sets as well as the hospitalization or physician visits fees before the patient's admission to the PICU, were not included.

Income of the family was interpreted based on the official lowest salary fixed by the government (XAF 90,000/USD 153.53). The income was low when it was lower than XAF 90,000/USD 153.53, middle when it was between XAF 90,000/USD 153.53 and XAF 500,000/USD 852.96, and high when it was above USD XAF 500,000/USD 852.96.

The data were computed on Microsoft Excel 2013 and processed and analyzed with the software STATA (version 12, Texas, USA). For the description of each quantitative variable, the mean/average value, median, and standard deviation were calculated. For the comparison of qualitative data, the ANOVA and Student tests were used. The tests were significantly significant when *p* value <0.05. The different costs have been calculated in XAF and then converted into USD using the exchange rate of USD 1 = XAF 568.19.

## 3. Results

### 3.1. Characteristics of the Population

1278 children were admitted in PICU during our study. Among them, 136 (10.64%) had sickle-cell disease and 94 (69.11%) had developed severe acute complications. These were 48 boys (51.1%) and 46 girls (48.9%) making a sex ratio of 1.04. the patients were aged from 6 to 192 months with an average age of 69 ± 50 months. Fifty-eight patients (61.7%) were coming to the hospital from their homes whereas 24 children (25.5%) were referred by primary and secondary health facilities. Twelve children (12.8%) were from other teaching hospital departments. It had been possible to determine the income of 50 families (53.2%). Among them, 26 (52%) had low status income while 18 (36%) had middle and 6 (12%) had high one (Table 1).

### 3.2. Costs of Hospital Expenses

The average length of hospitalization has been 5.5 days (extremes of 1 and 16 days) generating a median cost of XAF 30,000/USD 52.79 (range XAF 25,000/USD 42.64 and XAF 80,000/USD 136.47). Sixteen children (17%) died during their hospitalization. The mortality rate was significantly higher in the age group older than 120 months with no influence of referral origin of the patient (*p* = 0.004).

The median global cost care of SCD related acute complications was XAF 65.460/USD 111.67 (range XAF 28,305/USD 49.81 and XAF 365,740/USD 643.69). Diagnostic tests, hospitalizations, and medicines represented, respectively, 16%, 38%, and 49% of the global cost (Table 2).

Bacterial infections were the most frequent acute complication associated with SCD related crisis with 50 cases (53.2%). Care of bacterial infections whatever the type of sickle-cell crisis they were triggering was the most expensive since the quotation for their care was the highest (range: XAF 62,800/USD 107.13 and XAF 135271.5/USD 230.76), followed by malaria (range: XAF 28.305/USD 48.28 and XAF 99,944/USD 170.49). Vascular complications were represented by acute chest syndrome and stroke with respective costs care of XAF 42,800/USD 73.01 and XAF 103,492/USD 176.55 (*p* = 0.041) Table 3.

## 4. Discussion

This first study has allowed estimating the cost of care of severe acute complications SCD related in pediatric population admitted in intensive care. The global cost borne by families is high since it is on average XAF 65,460/USD 111.67 per episode, representing 2/3 of the minimum wage salary in Congo officially set at XAF 90,000/USD 153.53. Our study has several limits. First, the analysis of these results does not take into account the fees generated by consumables (syringes, infusion sets, and dressings). They are also to be paid by the patient, as well as fees for preadmission physician visit or hospitalization in different hospitals or units that concerned 38.3% of our sample population. The second limitation is the care costs estimations. Estimated costs were calculated from the teaching hospital costing that has the lowest fees. Nevertheless, fees displayed by the teaching hospital do not allow a real cost recovery, limiting a sustainable procurement in reagents but also in medicines. Referring to a WHO study, availability of medicines is limited. Only 60% of essential medicines are available in public pharmacies [8]. These parameters, which are difficult to evaluate since many medicines and tests are bought and used out of the teaching hospital, could contribute to underestimating the real care cost of SCD related acute complications. A similar study conducted on adults in the clinical hematology department reported a comparable average global cost care: XAF 88,365/USD 155.520 [12]. Nevertheless, some reservation must be expressed in comparing these costs, since the hospitalization fees in the clinical hematology department are fixed at XAF 10,000/USD 17.05 no matter the length of hospitalization. Consequently, the fees for hospitalization represented only 8.8% of the global expense in that study compared to 38% in ours [12].

Bacterial infections are the first cause of admission of sickle-cell disease children and adults in Africa [13–16]. The cost of care for their management is higher whatever the type of associated severe acute complications (XAF 66,765/USD 113.89 to XAF 135,271/USD 230.78). This is the consequence of the high cost of antibiotics in the continent [8, 17, 18]. Despite an ambitious essential medicines policy (with antibiotics in priority position) in Congo that aims to reduce the cost of medicines, these medicines remain inaccessible for the majority of the population [8]. Additionally, only 57% of antibiotics are prescribed by their international nonpropriety name (INN) [9]. This trend seems similar in sub-Saharan Africa as a study in private pharmacies in Mali showed that only 48.2% of antibiotics were prescribed in INN [18]. Also, procurement challenges at the hospital pharmacy, limited availability of essential medicines, and presence of fake antibiotics on the market are some elements pushing for prescription and purchase of branded medicines. Besides, microbiologic proof of infections barely provided by our laboratories is responsible of an overprescription of antibiotics and then an over cost care. Lastly, the absence of standard therapeutic chart has a perceptible impact on the cost of the prescription by irrational use of antibiotics.

Malaria represented the second cause of admission of sickle-cell disease children with a total of 29.8%. Malaria is widely considered a major cause of illness and death in SCD patients [14, 19, 20]. Even though we find in our study strong association between hyperhemolysis crisis and malaria, the association of this comorbidity is controversial. Some investigators have associated it with the anemic hyperhemolysis crisis while others suggest that patients living with SCD are protected from malaria [21, 22]. Despite the fact that the National Programme on Malaria has chosen the combination of artesunate and amodiaquine as first-line treatment, quinine is still used in first intention in almost 85% of health facilities [9].

The additional costs of the prescription in our study come from the purchase of pain killers and labile blood products. Blood transfusion is a major therapeutic element in the treatment of sickle-cell disease crisis since it is performed on 47% of sickle-cell disease children [14]. The supply cost for a bag of erythrocyte concentrate is XAF 7,500/USD 12.79, whereas the effective expenses for its preparation are estimated at XAF 65,000/USD 110.88. This causes supply challenges as mentioned earlier.

## 5. Conclusion

This study has examined the cost of severe acute SCD related complications in intensive care. Severe acute SCD related complications remain worrisome not only due to their graveness, but mainly due to the expenses that families must bear for their care. This study is the first to examine the cost of all components of care for pediatrics population with SCD admitted in intensive care. It is an important input to SCD treatment strategies, health care planning, and research prioritization. This study has also shown that infections and malaria remain persistently high. Additional research is needed to better understand infectious pattern of children with SCD.

## Figures and Tables

**Table 1:** Characteristics of the population.

*Gender*	*n* (%)
Female	46 (48.9)
Male	48 (51.1)
Sex ratio	1.04

*Age *(*months*)	
Mean ± Ecart type	69.26 ± 50.40
Min–max	6–192

*Referred by *	*n* (%)
Teaching hospital's departments	12 (12.8)
Primary and secondary public offices	14 (14.9)
Private offices	10 (10.6)
Home	58 (61.7)

*Hospitalization length *(*days*)	*n* (%)
1-2	44 (46.8)
3–8	34 (36.2)
9–16	16 (17)

*Income *	*n* (%)
Low	26 (27.7)
Middle	6 (6.4)
High	18 (19.1)
Unknown	44 (46.8)

*Death *	*n* (%)
Yes	78 (83.0)
No	16 (17.0)

*Age of death* ^*∗*^	*n* (%)
0–24	1 (12.5)
25–60	0 (00)
61–120	6 (37.5)
>120	8 (50)

^*∗*^ *p* = 0.004.

**Table 2:** Acute severe sickle-cell complications global treatment cost in XAF (USD).

	Hospitalization cost	Diagnosis test cost	Medicines cost	Global cost care
Mean	33,085.1 (58.23)	12,068.18 (21.23)	53,647.67 (94.41)	93,889.21 (165.24)

Ecart type	16,535.4 (29.1)	10,493.76 (18.46)	59,461.21 (104.65)	78,622.47 (138.37)

Median	30,000 (52.79)	10,500 (18.47)	32,267.5 (56.79)	65,460 (111.67)

Min–max	25,000–80,000 (42.64–136.47)	0–48,000 (0–84.47)	3,305–272,740 (5.81–480.02)	28,305–365,740 (49.81–643.69)

*t* = 2028, *p* = 0.026.

**Table 3:** Severe acute sickle-cell complications and its generated factors treatment cost.

Diagnostic	*n*	%	Global management care XAF (USD)	*p* value
Major acute pain + bacterial infections	12	12.8	66,765 (117.50)	

Hyperhemolysis + bacterial infections	18	19.2	103,492 (182.14)	*p* = 0.041

Mixed severe acute crisis + bacterial infections	28	29.8	135,271 (238.07)	

Major acute pain + malaria	4	4.2	28,305 (49.82)	

Hyperhemolysis + malaria	16	17,1	64,891.63 (114.21)	

Mixed severe acute crisis + malaria	8	8.5	99,944 (175.90)	

Major acute pain + acute chest syndrome	2	2.1	42,800 (75.33)	

Hyperhemolysis + acute chest syndrome	2	2.1	62,800 (110.52)	

Mixed severe acute crisis + stroke	4	4.2	130,333 (229.38)	

Total	94	100		
